# Intraperitoneal *Enterobius vermicularis* Infection: A Case Report

**DOI:** 10.1155/S1064744996000075

**Published:** 1996

**Authors:** Mehmet R. Gazvani, Simon J. Emery

**Affiliations:** 1 Departnemt of Obstetrics and Gynaecology Singleton Hospital Swansea United Kingdom; 2 Departnemt of Obstetrics and Gynaecology Liverpool Women's Hospital Crown Street Liverpool L 8 7 SS United Kingdom

## Abstract

*Background: Enterobius vermicularis* in an ectopic location usually represents an incidental finding in tissue specimens in a patient without symptoms. However, the parasite can lead to inflammation and symptoms in rare cases.

*Case:* A 36-year-old woman had an abdominal hysterectomy for menorrhagia, dyspareunia, and lower abdominal pain. Two small nodules from the posterior aspect of the left broad ligament as well as the uterus were sent for histologic examination. The pathologic findings confirmed the diagnosis of “granulomatous peritoneal nodules due to *E. vermicularis*.” Within the nodules were aggregates of ova of *E. vermicularis*.

*Conclusion:* This case reminds us that such granulomatous lesions can simulate leiomyoma, fibroma, endometrioma, and tuberculous or malignant lesions. It is important to be aware of this resemblance to avoid unnecessary surgical intervention.


Infectious Diseases in Obstetrics and Gynecology 4:28–30 (1996)
© 1996 Wiley-Liss, Inc.

### Intraperitoneal *Enterobius vermicularis* Infection: A Case Report

Mehmet R. Gazvani and Simon J. Emery

*Department of Obstetrics and Gynaecology, Singleton Hospital, Swansea, United Kingdom*

ABSTRACT

***Background:*** ***Enterobius vermicularis*** **in an ectopic location usually represents an incidental finding in tissue specimens in a patient without symptoms. However, the parasite can lead to inflammation and symptoms in rare cases.**

***Case:*** **A 36-year-old woman had an abdominal hysterectomy for menorrhagia, dyspareunia, and lower abdominal pain. Two small nodules from the posterior aspect of the left broad ligament as well as the uterus were sent for histologic examination. The pathologic findings confirmed the diagnosis of "granulomatous peritoneal nodules due to *E. vermicularis*." Within the nodules were aggregates of ova of *E. vermicularis.***

***Conclusion:*** **This case reminds us that such granulomatous lesions can simulate leiomyoma, fibroma, endometrioma, and tuberculous or malignant lesions. It is important to be aware of this resemblance to avoid unnecessary surgical intervention.** © 1996 Wiley-Liss, Inc.

KEY WORDS

*Oxyuris*, parasite, granuloma, pinworm

Granulomatous lesions caused by *Enterobius vermicularis* in ectopic locations are rare, generally representing incidental findings in surgical or autopsy tissue specimens in patients without known symptoms,[1] because of the low pathogenicity of *E. vermicularis.*[2] However, in a few cases, the parasite has provoked or set the stage for sufficient inflammatory reaction to cause symptoms. We present another unusual case in which we feel that the presence of the parasite in an ectopic location led to inflammation and symptoms.

### CASE REPORT

A 36-year-old woman presented as a gynecology outpatient with a history of longstanding dyspareunia, left-sided lower abdominal pain, and menorrhagia which had not responded to danazol.

Her clinical examination and findings were normal. Having previously had an unsuccessful transcervical endometrial resection for menorrhagia, she was scheduled for an abdominal hysterectomy.

During her routine hysterectomy, in which both ovaries were conserved, 2 small nodules on the posterior aspect of the left broad ligament were excised and sent for histologic evaluation.

The pathologic findings confirmed the diagnosis of "granulomatous peritoneal nodules due to *E. (Oxyuris) vermicularis.*" The specimen consisted of 2 firm nodules, the larger being 1.2 cm in maximum diameter. Microscopically, the nodules were partly fibrotic and partly necrotic, with central calcification and peripheral eosinophils and giant cells, lymphocytes, and histiocytes. Within the nodules were aggregates of ova of *E. vermicularis* (Fig. 1). There was no evidence of *E. vermicularis* infection in the uterus or cervix.

Six months later, the patient remained symptomatic, with minimal benefit from her hysterectomy.

Address correspondence/reprint requests to Dr. Mehmet R. Gazvani, Department of Obstetrics and Gynaecology, Liverpool Women's Hospital, Crown Street, Liverpool L 8 7 SS, United Kingdom.

Received April 1, 1996

Accepted April 29, 1996

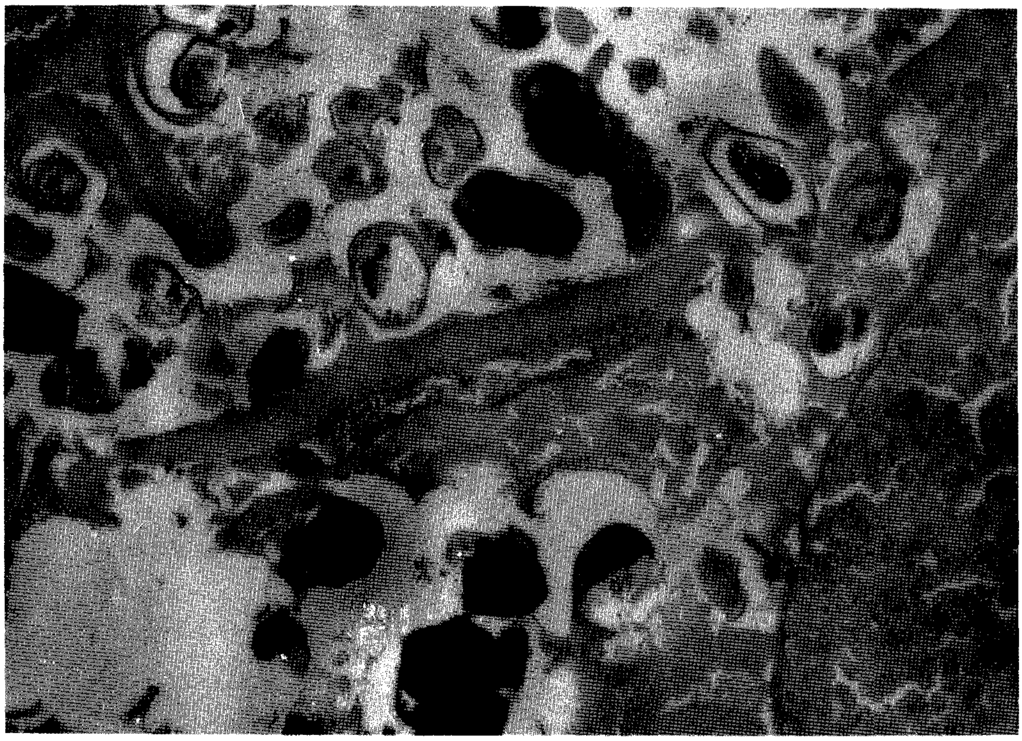

Fig. 1. Granulomas containing aggregates of ova of *E. vermicularis*. Hematoxylin-eosin. ×50.

Further investigation, including a laparoscopy and peritoneal biopsies, failed to indicate a definitive etiologic factor for her symptoms.

### DISCUSSION

*E. vermicularis* is the only nematode that infects man.[3] The mature pinworm inhabits the lumen of the intestine in the region of the lower-most part of the ileum, the cecum, the proximal part of the ascending colon, and the appendix. The gravid females migrate down the colon to the anogenital region where they lay eggs.[4]

It is generally accepted that the adults of *E. vermicularis* may travel from the perineum into the vagina and from the vagina to all levels of the female genital tract.[5] The worm (or its ova) has been found in the vagina, endometrium, myometrium, and fallopian tube. The parasite has also been found in the peritoneal cavity. As the latter usually occurs in women, it is presumed that the worm arrives at this site by migration through the fallopian tube.[2] In addition, pinworm granulomas have been found in the liver, lung, prostate, and renal pelvis.[4] The passage of *E. vermicularis* through the intestinal wall to produce pelvic peritoneal granulomas is another possible mechanism, but it is very difficult to prove because the infection is seldom found in the bowel wall.[6]

*E. vermicularis* granulomas are usually incidental findings that do not cause clinical problems. Occasionally, the lesions cause symptoms. In the case presented, although the vague abdominal pain and dyspareunia were present on the same side as the granulomas, the patient's symptoms did not improve following her hysterectomy and removal of the lesions. Possibly, the initial infection with *E. vermicularis* caused granulation and scarring in the pelvis that resulted in her presenting symptoms. In this case, no specific treatment was employed, as advised by the pathologists, who found no sign of active disease.

In the case presented, intraperitoneal *E. vermicularis* infection caused granulomatous peritoneal nodules. Granulomatous lesions can simulate leiomyoma, fibroma, endometrioma, and tuberculous or malignant lesions. Therefore, all infectious causes, including *E. vermicularis*, should be excluded in order to avoid unnecessary surgical intervention.

### REFERENCES

1. McCabe K, Nahn PAK, Sahin AA, Mitchell MF: Enterobiasis of the ovary in a patient with cervical carcinoma in situ. Infect Dis Obstet Gynecol 2:231–234, 1995.
2. Symmers W St C: Pathology of oxyuriosis with special reference to granulomas due to presence of *Oxyuris vermicularis (Enterobius vermicularis)* and its ova in tissues. Arch Pathol 50:475–516, 1950.
3. Neri A, Tadir Y, Grausbard G, Pardo J, Ovadia J, Braslavsky D: *Enterobius (Oxyuris) vermicularis* of the pelvic peritoneum—A cause of infertility. Eur J Obstet Gynaecol Reprod Biol 23:239–241, 1986.
4. Tsung SH, Loh WP: Invasion of the fallopian tube by *Enterobius vermicularis*. Ann Clin Lab Sci 9:393–395, 1979.
5. Saffos RO, Rhatigan RM: Unilateral salpingitis due to *Enterobius vermicularis*. Am J Clin Pathol 67:296–299, 1977.
6. Vinuela A, Fernandez-Rojo F, Martinez-Merino A: *Oxyuris* granulomas of pelvic peritoneum and appendicular wall. Histopathology 3:69–77, 1979.
